# Case report: Efgartigimod treatment in two pediatric patients with chronic inflammatory demyelinating polyneuropathy

**DOI:** 10.3389/fimmu.2025.1741057

**Published:** 2026-01-12

**Authors:** Yanping Ran, Wenlin Wu, Chi Hou, Haixia Zhu, Wenxiao Wu, Zongzong Chen, Xuexia Ma, Xiaolan Mo, Houliang Deng, Yuanyuan Gao, Xiaojing Li

**Affiliations:** 1 Department of Neurology, Guangzhou Women and Children’s Medical Center, Guangzhou Medical University, Guangzhou, Guangdong, China; 2 Department of Pharmacy, Guangzhou Women and Children’s Medical Center, Guangzhou Medical University, Guangzhou, Guangdong, China

**Keywords:** case report, children, CIDP, efgartigimod, treatment

## Abstract

**Background:**

Chronic inflammatory demyelinating polyneuropathy (CIDP) is the most common chronic immune-mediated polyneuropathy. Early and effective immunomodulatory treatment is essential to prevent long-term disability. Efgartigimod, a human IgG1 Fc fragment, has recently been approved for adult CIDP, but its safety and efficacy in pediatric CIDP remain unstudied. We retrospectively analyzed two pediatric patients with CIDP who received efgartigimod treatment.

**Case report:**

Age at onset was 1.5 and 3.4 years; disease duration before efgartigimod was 1.5 and 3.2 years; and age at initiation was 3.0 and 6.6 years. Prior treatments included corticosteroids, maintenance intravenous immunoglobulin, mycophenolate mofetil, and rituximab in one patient, and corticosteroids plus maintenance intravenous immunoglobulin in the other. Both initiated efgartigimod due to uncontrolled relapses. At final follow-up (8 months), both showed improvement in Medical Research Council (MRC) sum scores and Inflammatory Neuropathy Cause and Treatment (INCAT) scores. One patient experienced a single relapse; the other remained relapse-free. Two mild, self-limited adverse events occurred: upper respiratory tract infection in one and transient alanine aminotransferase elevation in the other.

**Discussion:**

This is the first report of efgartigimod use in pediatric patients with CIDP. Efgartigimod was generally well tolerated, and some clinical improvements were observed in this population. However, given the small sample size, these findings should be considered preliminary and require confirmation in larger, prospective studies.

## 1 Introduction

Chronic inflammatory demyelinating polyneuropathy (CIDP) is the most common chronic immune-mediated polyneuropathy, with a worldwide prevalence of approximately 3 per 100,000 (1). The prevalence rate of CIDP in children is 0.22 per 100,000 (2). CIDP typically presents as either a relapsing (61%) or progressive (39%) neuropathy with both proximal and distal weakness developing over a period of at least 8 weeks, and is characterized by symmetric paresthesia, weakness, and sensory dysfunction in the extremities (3). CIDP may also present with areflexia, cranial nerve involvement, autonomic symptoms, and neuropathic pain, although these features are less common (4, 5).

Timely initiation of immunomodulatory treatment are crucial to prevent long-term disability. No randomized controlled trials have been conducted in children with CIDP, and treatment strategies are largely extrapolated from adult data. First-line treatment for CIDP includes corticosteroids, intravenous immunoglobulin (IVIG) and plasma exchange. Steroid- or immunoglobulin-sparing agents such as azathioprine, mycophenolate mofetil (MMF), or ciclosporin may be considered for maintenance therapy. In refractory cases, cyclophosphamide, ciclosporin, or rituximab may be used (6). Despite these treatment options, a significant proportion of patients remain difficult to manage. Approximately 20%–30% of CIDP cases are treatment-resistant with a prolonged disease course, requiring long-term medication (6). These patients often respond poorly to current immunotherapies and face considerable drug-related side effects and treatment burdens. Recurrent relapses significantly impair quality of life and may result in severe neurological disability (7).

Efgartigimod, a neonatal Fc receptor (FcRn) antagonist, has emerged as a promising candidate in this context. It has been approved as an add-on therapy for adult patients with generalized myasthenia gravis (MG) (8). A recent multicenter, randomized-withdrawal, double-blind, placebo-controlled phase 2 trial (the ADHERE study) demonstrated that subcutaneous (SC) efgartigimod PH20 was effective and well tolerated in adults with CIDP, significantly reducing the risk of relapse compared to placebo (7). Efgartigimod has not been approved by the U.S. Food and Drug Administration (FAD) or the European Medicines Agency (EMA) for use in children with CIDP. Its use in pediatric CIDP has not been reported. This study aims to assess the safety and therapeutic potential of efgartigimod in children with CIDP.

## 2 Subjects and methods

### 2.1 Subjects

Children (younger than 18 years old) with CIDP who received efgartigimod treatment at the Department of Neurology of Guangzhou Women and Children’s Medical Center, Guangzhou Medical University, between December 2024 and July 2025 were included. The diagnosis of CIDP was based on the 2021 European Federation of Neurological Societies/Peripheral Nerve Society (EFNS/PNS) criteria (6).

### 2.2 Methods

The efgartigimod treatment regimen was adapted from adult CIDP and AChR-positive generalized MG protocols, with adjustments for the pediatric population (7, 9). An initial dose of 10 mg/kg was administered weekly for four weeks, followed by individualized maintenance infusions at intervals not exceeding 8 weeks, determined according to clinical response, relapse frequency, disease activity and serum IgG level. The 8-week upper limit was chosen based on pharmacodynamic data showing that serum IgG levels typically return to baseline approximately 8 weeks after the last dose, ensuring sustained immunomodulatory effect (10).

## 3 Case presentations

### 3.1 Case 1 (male, onset at 1.5 years old)

The patient presented with an acute onset of progressive, symmetric limb weakness lasting 10 days, which led to an inability to walk and raise his arms. His prior development was age-appropriate, with no personal or family history of neurological or autoimmune disorders. He was admitted to a local hospital. Cerebrospinal fluid (CSF) analysis revealed albuminocytologic dissociation, with elevated total protein (600 mg/dL) and a normal white blood cell count (WBC) (2 × 10^6^/L). Serum anti-GT1a IgG was positive. He was diagnosed with Guillain-Barré syndrome and treated with IVIG (2 g/kg over 5 days), resulting in full recovery to his pre-onset baseline. Two months after onset, he developed recurrent symmetric lower limb weakness, again rendering him unable to walk. Spinal magnetic resonance imaging (MRI) with gadolinium contrast revealed mild thickening and enhancement of the cauda equina nerves. He received IVIG (2 g/kg), after which limb strength recovered. At three months, he experienced another episode of symmetric limb weakness with inability to walk or lift his arms. Electrophysiological studies revealed a demyelinating polyneuropathy with secondary axonal involvement, characterized by prolonged distal latencies, markedly reduced compound muscle action potential (CMAP) amplitudes, absent F-waves, abnormal temporal dispersion, and absent sural sensory nerve action potentials (SNAPs), predominantly affecting the lower limbs. A third IVIG course (2 g/kg) led to complete neurological recovery. He subsequently experienced relapses at 4.0, 4.6, and 5.6 months after onset, each marked by an inability to walk and raise both arms, occasionally accompanied by hypophonia. The episode at 4.6 months was additionally managed with oral prednisone (2 mg/kg). These relapses were treated with IVIG at doses of 1 g/kg, 2 g/kg, and 2 g/kg, respectively, with full recovery achieved after each course.

**Figure 1:**
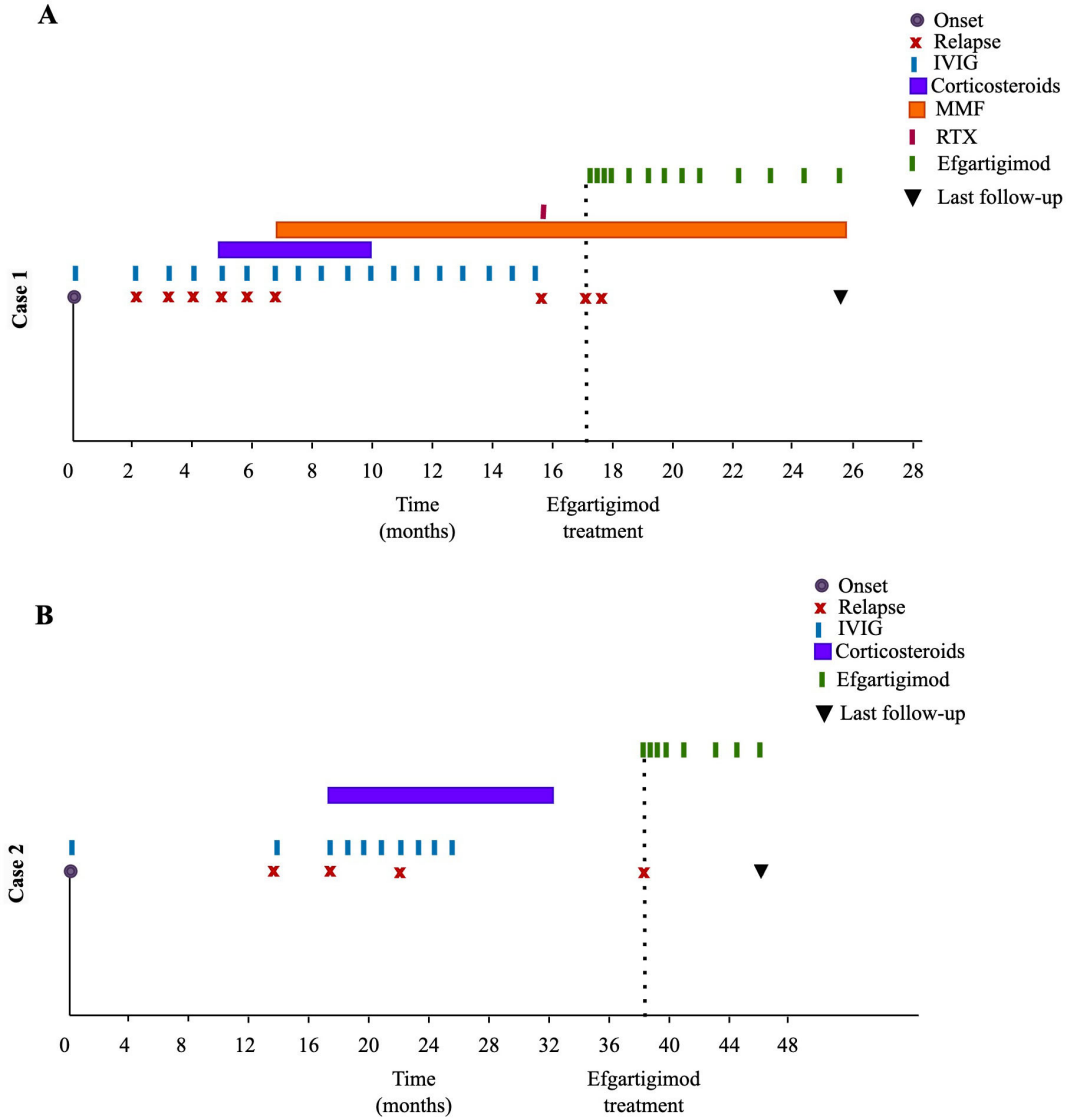
Disease course and treatment regimens in two paediatric patients with CIDP. **(A)** Timeline of disease progression and immunotherapy in Case 1. **(B)** Timeline of disease progression and immunotherapy in Case 2. IVIG, intravenous immunoglobulin; MMF, mycophenolate mofetil; RTX, rituximab.

After that, he was transferred to our hospital. On admission to our hospital, physical examination revealed that muscle strength was graded as Muscle strength was graded as Medical Research Council (MRC) grade III proximally and III− distally in all four limbs, with decreased muscle tone. Deep tendon reflexes were absent. Serum complement (C3, C4) and serum multiplex cytokine panel (included TNF-β, IL-12p70, IL-1β, IL-10, IL-6, TNF-α, IL-2, IFN-γ, IL-17F, IL-8, IL-4, IL-5, IL-17A, and IL-22) were within normal ranges. CSF analysis revealed albuminocytologic dissociation, with elevated total protein (600 mg/dL) and a WBC count of 13 × 10^6^/L. CSF multiplex cytokine panel showed elevated interleukin-8 (26.11pg/ml, normal range: 0~15.71pg/ml). Spinal MRI with gadolinium contrast showed mild thickening and enhancement of the spinal nerve roots below the T11 vertebral level. Whole-exome sequencing and mitochondrial genome testing revealed no pathogenic or likely pathogenic variants relevant to the patient’s clinical presentation. The patient was diagnosed with CIDP and treated with intravenous methylprednisolone (IVMP, 15 mg/kg), followed by a tapering course of oral prednisone (2 mg/kg), in combination with IVIG (2 g/kg), resulting in full recovery. At 6.6 months after onset, the patient experienced a relapse. He was treated with IVIG (2 g/kg), after which MMF was initiated. Maintenance IVIG (1 g/kg) was administered every three weeks thereafter. At 15.5 months after onset (three days after the last IVIG treatment), he experienced a relapse, presenting with inability to walk. The paranodal antibody panel (including neurofascin-155 IgG4, neurofascin-186 IgG, contactin-1 IgG4, contactin-2 IgG, and contactin-associated protein-1 IgG, tested by cell-based assays) and serum testing for anti-ganglioside antibodies were negative. He received immunosuppressive therapy with rituximab (RTX) at a dose of 375 mg/m² once weekly for four weeks. Following treatment, his symptoms improved and peripheral blood CD19^+^ B-cell count was depleted to 0.00 cells/μL. However, three days after completing the RTX course—one day prior to admission—he experienced a relapse of limb weakness, which improved after treatment with IVIG (1 g/kg) (**Figure 1A**).

**Figure 2:**
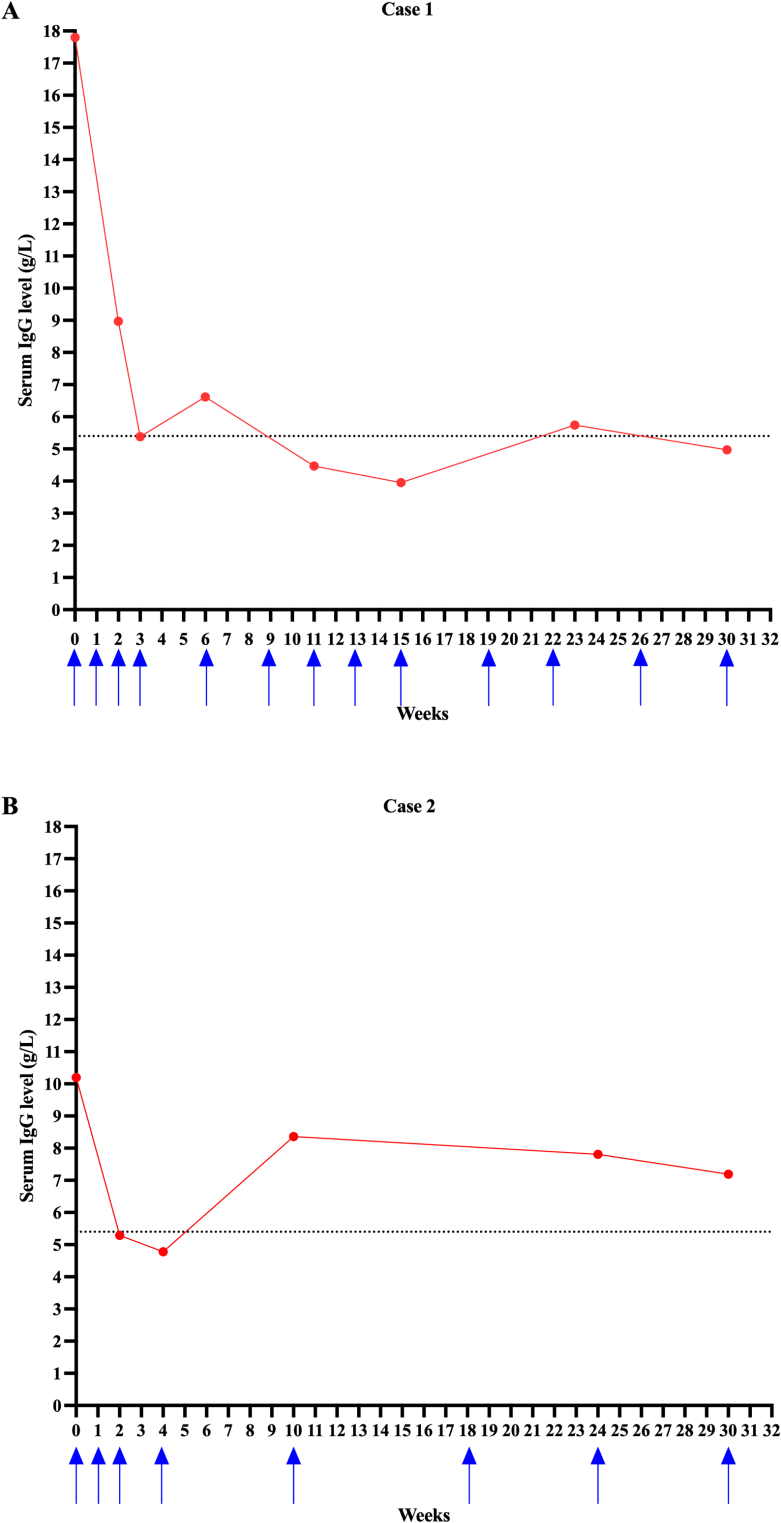
Serum IgG levels in two paediatric patients with CIDP receiving efgartigimod treatment. **(A)** Longitudinal serum IgG concentrations in Case 1. **(B)** Longitudinal serum IgG concentrations in Case 2. Blue arrows indicate the timing of efgartigimod administration. The dashed horizontal line represents the lower limit of the normal IgG range (5.4 g/L).

At 17.2 months after onset, the patient experienced a relapse, presenting with inability to walk and raise his arms. Physical examination revealed that muscle strength was graded as Muscle strength was graded as Medical Research Council (MRC) grade III proximally and III− distally in all four limbs, with decreased muscle tone. Deep tendon reflexes were absent. B-cell depletion persisted, with a CD19^+^ B-cell count of 1.00 cells/μL. The MRC sum score was 18, and the Inflammatory Neuropathy Cause and Treatment (INCAT) disability score was 8 (4 for the upper limbs and 4 for the lower limbs). The CIDP Disease Activity Status (CDAS) score was 5C. Efgartigimod treatment was subsequently initiated at the age of 3.0 years old, resulting in clinical improvement. By week 2, the MRC sum score had improved to 56, and the INCAT disability score had decreased to 3 (2 for the upper limbs and 1 for the lower limbs). Following the second efgartigimod cycle, the patient developed a mild upper respiratory tract infection and experienced a relapse, with the MRC sum score declining to 18 and the INCAT disability score increasing to 8 (4 for the upper limbs and 4 for the lower limbs). Symptomatic treatment was administered, and no further relapses occurred thereafter. By week 4, the MRC sum score had again improved to 56, and the INCAT score decreased to 3 (2 for the upper limbs and 1 for the lower limbs) (**Table 1**). At the last follow-up, after 8 months of continuous treatment, the MRC sum score remained stable at 56, and the INCAT disability score further improved to 1 (upper limb: 1). Following efgartigimod administration, serum IgG levels progressively declined and dropped below the lower limit of the normal reference range (5.4 g/L) after the loading dose phase. A transient rise in IgG concentrations was observed during extended dosing intervals (details shown in **Figure 2A**). No serious or severe adverse events were reported. Despite the transient drop in IgG levels below the normal lower limit during the third week, the patient experienced a mild upper respiratory tract infection and relapse at that time. However, in the subsequent treatment phase, during which IgG levels remained below the normal lower limit, the patient did not experience any further infections, relapses, or disease fluctuations.

**Table 1:** Characteristics of the patients.

Patient	Case 1	Case 2
Age at onset (yrs)	1.5	3.4
Sex	Male	Male
Clinical manifestations	Recurrent symmetric limb weakness over 8 weeks	Recurrent symmetric proximal and distal muscle weakness over 8 weeks
Serum complement		
C3 (g/L, normal range: 0.8-1.5g/L)	1.12	0.83
C4 (g/L, normal range: 0.13-0.43g/L)	0.14	0.19
Multiplex cytokine panel (included TNF-β, IL-12p70, IL-1β, IL-10, IL-6, TNF-α, IL-2, IFN-γ, IL-17F, IL-8, IL-4, IL-5, IL-17A, and IL-22)		
serum	Normal	Normal
CSF (normal range of IL-8: 0~15.71pg/ml)	IL-8 26.11pg/ml, other cytokine markers within normal range	IL-8 22.15pg/ml, other cytokine markers within normal range
CSF analysis		
WBC count( 10^6^/L, normal range: 0~15)	13	2
Total protein(mg/dL, normal range:0~450)	600	480
Spinal MRI	Mild thickening and enhancement of the spinal nerve roots below the T11 vertebral level	Mild enhancement of the cauda equina nerves
Age of receiving efgartigimod treatment (yrs)	3.0	6.6
Weight at efgartigimod initiation (kg)	12.0	26.9
Disease duration prior to efgartigimod initiation (yrs)	1.5	3.2
Immunotherapy before efgartigimod treatment	Corticosteroids, maintenance IVIG, MMF and RTX	Corticosteroids, maintenance IVIG
Reason for efgartigimod treatment	Persistence of relapse	Persistence of relapse
Duration of efgartigimod treatment (mo)	8	8
Relapse during efgartigimod treatment	Yes	No
Physical examination before efgartigimod treatment	MRC grade III proximally and III− distally in all four limbs, with decreased muscle tone. Deep tendon reflexes were absent.	MRC grade V- proximally and IV distally in all four limbs, with decreased muscle tone. Deep tendon reflexes were reduced.
Physical examination after efgartigimod treatment	MRC grade V proximally and IV+ distally in all four limbs, with decreased muscle tone. Deep tendon reflexes were absent.	MRC grade V proximally and IV+ distally in all four limbs, with decreased muscle tone. Knee-jerk reflex were reduced, the other deep tendon reflexes were normal.
MRC sum score before efgartigimod treatment	18	50
MRC sum score after efgartigimod treatment	56	56
INCAT disability score before efgartigimod treatment	8	1
INCAT disability score after efgartigimod treatment	3	1
Follow-up duration (mo)	8	8
Adverse effect of efgartigimod treatment	Mild upper respiratory tract infection	Transient ALT elevation

ALT, alanine aminotransferase; C3, Complement 3; C4, Complement 4; CMAP, compound muscle action potential; EMG, Electromyography; INCAT, Inflammatory Neuropathy Cause and Treatment; IVIG, intravenous immunoglobulin; IL, Interleukin; MMF, mycophenolate mofetil; mo, moths; MRC, Medical Research Council; MRI, magnetic resonance imaging; RTX, rituximab; SNAP, sural sensory nerve action potentials; yrs, years.

### 3.2 Case 2 (male, onset at 3.4 years old)

According to parental recall, the patient developed generalized limb weakness following a pneumonia episode. His prior development was age-appropriate, with no personal or family history of neurological or autoimmune disorders. He was admitted to a local hospital and received IVIG (total dose of 2.5 g), after which his symptoms resolved. At 14 months after onset, the patient developed progressive limb weakness over one month following a Coronavirus Disease 2019 infection, characterized by impaired extension of both hands and difficulty climbing stairs. He was admitted to our hospital. Physical examination revealed decreased muscle tone, with MRC grade III strength in the bilateral upper limbs and grade IV in the bilateral lower limbs. Deep tendon reflexes were absent. Serum complement and serum multiplex cytokine panel were within normal ranges. CSF analysis showed mildly elevated total protein (480 mg/dL) with a WBC of 2 × 10^6^/L. CSF multiplex cytokine panel showed elevated interleukin-8 (22.15pg/ml). Spinal MRI with gadolinium contrast revealed no remarkable abnormalities. A diagnosis of Guillain-Barré syndrome was made. The patient was treated with IVIG (2 g/kg). Following treatment, muscle strength improved to MRC grade IV in the upper limbs and grade V in the proximal lower limbs. After discharge, the patient underwent electrophysiological examination at another hospital, as this test was not available at our institution. Electromyography demonstrated a demyelinating polyneuropathy with axonal involvement, characterized by prolonged distal latencies, reduced conduction velocities, markedly decreased CMAP amplitudes, prolonged or absent F-waves, and absent or low-amplitude SNAPs with reduced sensory conduction velocities. At 17.3 months after onset, the patient presented with an inability to walk and raise his arms following an upper respiratory tract infection 10 days earlier. CSF analysis showed mildly elevated total protein (550 mg/dL) with a WBC of 5 × 10^6^/L. Spinal MRI with gadolinium contrast revealed mild enhancement of the cauda equina nerves. He was diagnosed with CIDP and treated with IVMP (10 mg/kg), followed by a tapering course of oral prednisone (2 mg/kg), in combination with IVIG (2 g/kg), resulting in full recovery. At 22.3 months after onset, the patient experienced a relapse, presenting with difficulty climbing stairs and finger weakness, with inability to lift heavy objects. The patient received IVMP (10 mg/kg), followed by a tapering course of oral prednisone (2 mg/kg), in combination with IVIG (2 g/kg), resulting in full recovery. Maintenance IVIG (400 mg/kg) was administered monthly for 7 months (**Figure 1B**), after which it was discontinued by the patient’s parents without medical consultation.

At 38 months after onset, the patient experienced a relapse, presenting with limb weakness. Whole-exome sequencing revealed no pathogenic or likely pathogenic variants relevant to the patient’s clinical presentation. The paranodal antibody panel testing and serum testing for anti-ganglioside antibodies were negative. The MRC sum score was 50, and the INCAT disability score was 1(upper limbs:1). The CDAS score was 5C. Efgartigimod treatment was initiated at the age of 6.6 years, resulting in clinical improvement. At the last follow-up, after 8 months of continuous treatment, the MRC sum score increased to 56, and the INCAT disability score remained stable at 1 (upper limb: 1) (**Table 1**), with no further relapse occurring. Serum IgG levels declined over the course of treatment and fell below the lower limit of normal following the initial loading phase. A temporary increase in IgG levels was noted during extended dosing intervals (details shown in **Figure 2B**). Although the patient’s IgG levels fell below the normal lower limit during the second week of treatment, no infections, relapses, or disease fluctuations were observed at that time. Additionally, during the subsequent treatment period, including when IgG levels remained below the normal lower limit, no further infections, relapses, or disease fluctuations occurred. Approximately six months after initiating efgartigimod treatment, the patient developed a mild elevation in alanine aminotransferase (ALT 50 U/L; normal range: 7–30 U/L). Oral glucuronolactone was administered, and two months later, repeat testing showed normalization of ALT levels. No infections or life-threatening injection-related reactions were reported.

## 4 Discussion

We retrospectively evaluated the off-label use of efgartigimod in two pediatric patients with CIDP. Prior to efgartigimod initiation, one patient had received corticosteroids, maintenance IVIG, MMF and RTX while the other had been treated with corticosteroids and maintenance IVIG. Efgartigimod was started due to recurrent relapses despite previous immunotherapy, with a treatment duration of 8 months in both cases. Following efgartigimod administration, both patients showed improvement in the MRC sum score. The INCAT disability score improved in one patient and remained stable in the other compared to baseline prior to treatment. One patient experienced a single relapse during treatment, whereas the other remained relapse-free.

Approximately 16% of CIDP cases present with acute symptom onset and reach their nadir within 8 weeks, thus classified as acute-onset CIDP (A-CIDP), a phenotype that can resemble Guillain–Barré syndrome (GBS). Although GBS is usually monophasic, 2–5% of patients experience recurrences—defined as at least two episodes with a nadir within 4 weeks and separated by ≥4 months when recovery is incomplete or ≥2 months when recovery is complete or near complete (11). Recurrent GBS, in contrast to A-CIDP, is characterized by distinct acute episodes separated by long asymptomatic intervals, with each episode reaching nadir within 4 weeks and showing no chronic progressive or stepwise deterioration. In A-CIDP, however, patients typically exhibit either continuous progression beyond 8 weeks or repeated deteriorations in close temporal proximity, reflecting a chronic immune-mediated demyelinating process rather than discrete relapses. This distinction is clinically relevant because recurrent GBS generally does not require long-term immunosuppression, whereas A-CIDP does.

Notably, 2–5% of patients initially diagnosed with GBS are ultimately reclassified as A-CIDP. Both of our patients were first diagnosed with GBS, underscoring the importance of early differentiation, as A-CIDP requires long-term immunomodulatory treatment. Based on the literature reviewed in Differentiating recurrent Guillain–Barré syndrome and acute-onset chronic inflammatory polyneuropathy: literature review (Inan et al., 2024), distinguishing A-CIDP from GBS is challenging, particularly in the early stages, due to overlapping features (11). Clinically, A-CIDP patients tend to be older at disease onset, present with prominent sensory symptoms, and have a history of type 2 diabetes more frequently. In contrast, GBS patients have a faster symptom onset, normal CSF proteins early on, and a higher incidence of antecedent infections. Additionally, the time from neurological symptoms to maximum disability is often less than 4 weeks in GBS, while it exceeds 8 weeks in CIDP. Electrophysiological studies reveal that acute inflammatory demyelinating polyneuropathy (AIDP) patients often show reduced superexcitability and prolonged relative refractory periods, while A-CIDP exhibits early repolarization patterns. Although sural-sparing patterns and A waves have been proposed as potential markers, studies report conflicting results. Nerve conduction blocks may appear in AIDP patients after one year, indicating ongoing demyelination, while A-CIDP may develop these blocks later. Ultrasonography, using the Bochum ultrasound score has shown higher sensitivity and specificity for A-CIDP, with enlarged sensory nerves typically seen in A-CIDP but not in GBS. Immunologically, A-CIDP is characterized by impaired Fas-mediated T-cell apoptosis, while GBS does not show this abnormality. Higher CSF IL-8 levels are also more common in GBS, with a cutoff value of 70 pg/mL suggested for differentiation. Additionally, some AIDP patients continue to meet CIDP criteria months to years after the acute episode, making the diagnosis more complex. These findings highlight the importance of combining clinical, electrophysiological, ultrasonographic, and immunological data for accurate diagnosis.

These cases raise further consideration of the underlying immune mechanisms and current treatment strategies in CIDP. CIDP is an immune-mediated disorder, though its exact pathogenesis remains unclear. Various immune components—autoreactive T and B cells, cytokines, complement factors, and autoantibodies—have been implicated. IgG autoantibodies are thought to contribute to disease mechanisms (12). Passive transfer of patient IgG in animal models induces demyelination and conduction block, and the efficacy of plasma exchange supports a humoral role (7). While no specific pathogenic autoantibody is identified in most patients, antibody binding to myelin and Schwann cells has been observed. Notably, antibodies targeting nodal and paranodal proteins (e.g., NF155, CNTN1, CASPR1, NF140, NF186) are present in about 10% of CIDP cases (12). Efgartigimod is a human IgG1 Fc fragment that targets the FcRn, blocking the recycling of IgG by binding to FcRn, thereby reducing circulating IgG levels—a mechanism believed to underlie its therapeutic effect in autoimmune diseases (12). In the ADHERE study—a multicentre, randomised-withdrawal, double-blind, placebo-controlled phase 2 trial evaluating SC efgartigimod PH20 in adults with CIDP—66% (214/322) of patients with active or worsening disease achieved clinical improvement during stage A. In stage B, efgartigimod significantly reduced the risk of relapse compared to placebo (hazard ratio 0.39 [95% CI 0.25–0.61]) (12). Based on these findings, SC efgartigimod PH20 received regulatory approval for adult CIDP in multiple regions. In China, it was approved by the National Medical Products Administration on November 11, 2024. To date, no clinical studies have reported efgartigimod use in paediatric CIDP. Our findings from two pediatric patients with active disease and suboptimal response to prior immunotherapies, who were treated with efgartigimod, showed improvements in MRC scores and reduction in serum IgG levels, consistent with FcRn-mediated IgG clearance. However, relapse during treatment in one patient—despite B-cell depletion—suggests that non-IgG-mediated immune mechanisms, such as T-cell activation or complement involvement, may also contribute to disease activity. These cases highlight the immunological heterogeneity of CIDP and underscore the need for biomarkers to guide FcRn-targeted therapy.

Efgartigimod, as a natural ligand of the FcRn, has attracted increasing interest in the treatment of neurological autoimmune diseases. Although some treatment regimens are shared across different diseases and trials, variations exist in dosing schedules and maintenance strategies, as outlined in **Table 2**. In the ADAPT study—a multicentre, randomised, placebo-controlled phase 3 trial evaluating intravenous efgartigimod in adults with MG, efgartigimod was administered at a dose of 10 mg/kg once weekly for four consecutive weeks per treatment cycle. Subsequent cycles could begin no earlier than 8 weeks from the initiation of the previous cycle (9). While in the ADHERE study, SC efgartigimod was administered at a dose of 1000 mg weekly (7). The first clinical trial of efgartigimod in children—targeting paediatric generalized MG-is currently in the recruitment phase. During the initial 8-week period, the study aims to evaluate the pharmacokinetics, pharmacodynamics, and safety of intravenous efgartigimod in this population. Given the limited data on efgartigimod use in children, the treatment regimen in our study was adapted from adult CIDP and MG protocols (7, 9). An initial dose of 10 mg/kg was administered weekly for four weeks. The two patients in our study, aged 3.0 and 6.6 years, subsequently received maintenance infusions at intervals guided by clinical response, relapse frequency, disease activity, and serum IgG levels. Infusion intervals did not exceed 8 weeks, as serum IgG levels typically return to baseline approximately 8 weeks after the last dose, thereby maintaining a sustained immunomodulatory effect (10). Both patients exhibited dynamic reductions in serum IgG levels during efgartigimod therapy, with transient increases observed during extended dosing intervals (**Figure 2**). Serum IgG levels declined by 69.8% and 53.1%, respectively, after the fourth infusion. In the ADAPT study, serum IgG levels were reduced by approximately 57.6% one week after the fourth infusion in the first treatment cycle, with levels returning to baseline by week 9 post-treatment (9). These results are consistent with the pharmacodynamic profile of efgartigimod and its proposed mechanism of action. Currently, pediatric pharmacokinetic and pharmacodynamic data for efgartigimod are limited. Ongoing clinical trials (e.g., NCT06392386) are specifically designed to assess the appropriate dosing regimen for pediatric populations. Future studies will help address dosing adjustments based on plasma volume differences and further evaluate the safety and infection risk profiles associated with IgG reduction in children.

**Table 2:** Clinical trials of efgartigimod in neurological diseases.

Drug (s)	Indication	Phase	Status	Treatment regimen	Identifier
Efgartigimod (IV)	MG in adults	III	Completed	10 mg/kg weekly ×4; ≥8 weeks from cycle start; 3-week infusion + 5-week follow-up	NCT03669588
Efgartigimod (IV)	MG in adults	III	Completed		NCT03770403
Efgartigimod (IV)	MG in adults	II/III	Active, not recruiting	Continuous Regimen Group:10 mg/kg every 2 weeks (q2w); Cyclic Regimen Group:10 mg/kg weekly ×4 per TP, 2 TPs with fixed 4-week interval between TPs	NCT04980495
Efgartigimod (IV)	MG in children	II/III	Recruiting	8-week dose-confirmatory phase (pediatric dosing under evaluation)	NCT04833894
Efgartigimod (IV)	MG in adults	II	Completed	Dose regimen not specified	NCT02965573
Efgartigimod (SC)	CIDP in adults	II	Completed	1000 mg, weekly	NCT04281472
Efgartigimod (SC)	CIDP in adults	II	Active, not	1000 mg, weekly	NCT04280718
Efgartigimod (IV)	MG exacerbation or crisis in adults	IV	Recruiting	10 mg/kg on days 1, 4, 11 and 18	NCT06860633
Efgartigimod (IV)	Guillain-Barré Syndrome in adults	II/III	Not yet recruiting	20 mg/kg on day 1 and day 5	NCT06885762
Efgartigimod (IV)	Guillain-Barré Syndrome in adults	II	Recruiting	20 mg/kg on day 1 and day 5	NCT05701189
Efgartigimod (IV)	Acute NMOSD in adults	Observational study	Not yet recruiting	10 mg/kg weekly ×4	NCT06118398

CIDP, chronic inflammatory demyelinating polyneuropathy; IV, intravenous; MG, myasthenia gravis; NMOSD, neuromyelitis optica spectrum disorder; SC, subcutaneous; TP, treatment period.

A comprehensive pooled analysis of global clinical trials—Safety profile of efgartigimod from global clinical trials across multiple immunoglobulin G-mediated autoimmune diseases (Gwathmey et al.)—demonstrated that, despite substantial reductions in total IgG, the incidence of severe or serious infections was comparable between efgartigimod- and placebo-treated participants (13). Most infections were mild to moderate, and no increase in infection rates was observed with long-term treatment. Notably, nadir IgG levels were not associated with infection risk, suggesting that FcRn blockade–mediated IgG reduction does not inherently predispose patients to infectious complications. These findings are consistent with our observations, in which neither patient experienced infections or relapses during periods of low IgG levels. In our study, both children exhibited transient declines in IgG below the lower limit of normal. In case 1, a mild upper respiratory tract infection and a relapse occurred when IgG was low during week 3; however, during subsequent treatment—despite persistently low IgG—no further infections or relapses occurred. In case 2, although IgG levels also fell below the normal lower limit during week 2, the patient remained clinically stable without infections, relapses, or disease fluctuations throughout follow-up. These findings suggest that short-term reductions in total IgG do not necessarily correlate with increased infection risk or clinical deterioration. Supporting this, previous studies indicate that infection risk becomes clinically relevant primarily when IgG levels fall below ~1 g/L for a sustained period—an effect rarely observed with efgartigimod (13, 14).

However, optimal dosing strategies in pediatric patients remain to be defined and require validation in prospective clinical studies. Efgartigimod is available in two formulations: intravenous (IV) and SC efgartigimod PH20. The SC formulation was evaluated in the ADAPT-SC study, a phase 3 randomized noninferiority trial in patients with MG, with long-term data from the ADAPT-SC+ open-label extension. ADAPT-SC demonstrated that SC efgartigimod PH20 (1000 mg) was noninferior to IV efgartigimod (10 mg/kg) in efficacy, with favourable long-term safety, tolerability, and sustained clinical benefit in a broad gMG population (15). Although SC efgartigimod PH20 was used in adults with CIDP in the ADHERE study, the IV formulation was selected for our pediatric patients for several reasons. First, economic considerations are particularly relevant in low-body-weight children. While both formulations allow for weight-based dosing, the IV formulation (400 mg/vial, ¥5,608 per vial) allows partial vial use, enabling cost-effective administration. In contrast, the SC formulation (1000 mg/vial, ¥20,772 per vial) has a higher unit cost and may be less economical for small-dose usage. Second, SC efgartigimod PH20 was not yet available in China when treatment was initiated in our patients, as it only became accessible on December 9, 2024.

In the ADAPT study, adverse events occurred in 77% of patients treated with efgartigimod and 84% of the placebo group. The most common adverse events included headache, nasopharyngitis, diarrhea, and upper respiratory or urinary tract infections, with a higher incidence of upper respiratory (11%) and urinary tract infections (10%) reported in the efgartigimod group compared to placebo (9). No clinically meaningful changes in hematology or chemistry parameters were observed in either group. In our study, one patient developed a mild upper respiratory tract infection, and the other exhibited a mild, transient ALT elevation not exceeding twice the upper limit of normal, which resolved with supportive care. According to the Common Terminology Criteria for Adverse Events version 5.0, this corresponds to a Grade 1 event. These findings were broadly consistent with previously reported safety profiles in adults, suggesting that intravenous efgartigimod may be tolerable in paediatric patients, although continued monitoring and further investigation are warranted.

This study has several limitations. First, it is a retrospective report of two individual cases without a control group, which limits the generalizability of these findings. Second, the follow-up duration was relatively short in some patients, making it difficult to assess long-term safety, sustained efficacy, and delayed adverse events. Third, a limitation of this study is the lack of longitudinal neurophysiological monitoring, as repeated nerve conduction studies and EMG were not performed due to limited access to these tests. Fourth, the decision to initiate efgartigimod treatment was made based on clinical judgment rather than standardized criteria, potentially introducing selection bias. Therefore, while the observed outcomes are encouraging, they should be interpreted with caution, and no definitive conclusions can be drawn regarding the efficacy, safety, or tolerability of efgartigimod in pediatric CIDP at this stage.

## 5 Conclusion

This is the first report of efgartigimod use in pediatric patients with CIDP. Efgartigimod was generally well tolerated and some clinical improvements were observed in this population. However, given the small sample size, these findings should be considered preliminary and require confirmation in larger, prospective studies.

## Data Availability

The original contributions presented in the study are included in the article/**Supplementary Material**. Further inquiries can be directed to the corresponding author.

## Supplementary material

Supplementary Table 1 **(A)** Electromyogram results of Case 1. **(B)** Electromyogram results of Case 2.

Supplementary Table 2 The standardized diagnostic criteria for CIDP in 2 children.
