# Supplementary material for: Case report: Efgartigimod treatment in two pediatric patients with chronic inflammatory demyelinating polyneuropathy
Source: Front Immunol. 2026 Jan 12;16:1741057. doi: 10.3389/fimmu.2025.1741057 (PMC12832919; doi:10.3389/fimmu.2025.1741057)
Supplement: Supplementary Table 1 — (A) Electromyogram results of Case 1. (B) Electromyogram results of Case 2. [file Table1.docx]

**Supplementary material**

**Table 1A** Electromyogram results of Case 1.

| Nerve | | Muscle/Segment | Latency (ms) | Amplitude | Velocity  (m/s) | F wave latency (ms) | % F |
| --- | --- | --- | --- | --- | --- | --- | --- |
| Motor NCS | Left common peroneal nerve | Ankle - EDB | 4.71 | 2.7 |  | — | — |
|  |  | B.Fibular head - Ankle | — | — | — |  |  |
|  | Left tibial nerve | Ankle - AH | 5.91 | 1.08 |  | — | — |
|  |  | Popliteal fossa - Ankle | — | — | — |  |  |
|  | Left median nerve | Wrist - APB | 4.69 | 1.1 |  |  |  |
|  | Right common peroneal nerve | Ankle - EDB | 5.97 | 1.56 |  | — | — |
|  |  | B.Fibular head - Ankle | — | — | — |  |  |
|  | Right tibial nerve | Ankle - AH | 6.09 | 0.87 |  | — | — |
|  |  | Popliteal fossa - Ankle | — | — | — |  |  |
| Sensory NCS | Left superficial peroneal nerve | Lower leg - Ankle | — | — |  |  |  |
|  | Left peroneal nerve | Lower leg - Ankle | — | — |  |  |  |
|  | Right superficial peroneal nerve | Lower leg - Ankle | — | — |  |  |  |
|  | Right peroneal nerve | Lower leg - Ankle | — | — |  |  |  |

Description:

SCV, Bilateral superficial peroneal nerves and sural nerves show sural sensory nerve action potentials (SNAPs) amplitude unable to be elicited.

MCV, Bilateral tibial nerves and distal common peroneal nerves show decreased compound muscle action potentials (CMAPs) amplitude, with proximal CMAP amplitude unable to be elicited. Bilateral tibial nerve CMAP waveforms exhibit marked dispersion.

F wave: Bilateral tibial nerves and common peroneal nerves: F waves cannot be elicited. Bilateral tibial nerves: M waves are scattered.

Conclusion: Peripheral nerve damage in both lower limbs, primarily demyelinating, affecting both motor and sensory functions.

**Table 1B** Electromyogram results of Case 2.

| Nerve | | Muscle/Segment | latency (ms) | Amplitude | Velocity  (m/s) | F wave latency (ms) | % F |
| --- | --- | --- | --- | --- | --- | --- | --- |
| Motor NCS | Left common peroneal nerve | Ankle-EDB | — | — |  |  |  |
|  |  | B.knee - ankle | 6.77 | 0.21 | — |  |  |
|  |  | A.knee - B.knee | 8.23 | 0.19 | 34.2 |  |  |
|  | Left tibial nerve | Ankle - AH | 13.5 | 0.43 |  |  |  |
|  |  | Knee - ankle | 35 | 0.2 | 11.4 |  |  |
|  | Left median nerve | Wrist - APB | 8.66 | 3.1 |  | — | — |
|  |  | B.Elbow - Wrist | 20.7 | 0.95 | 12.0 |  |  |
|  |  | Axilla - B.Elbow | 26.0 | 0.97 | 14.2 |  |  |
|  | Left ulnar nerve | Wrist - ADM | 6.61 | 4.8 |  | 80.0 | 84.4 |
|  |  | B.Elbow - Wrist | 16.1 | 0.69 | 10.5 |  |  |
|  |  | A.Elbow - B.Elbow | 22.3 | 0.95 | 14.5 |  |  |
|  |  | Axilla - Elbow | 24.5 | 0.61 | 25.0 |  |  |
|  | Right median nerve | Wrist - APB | 7.84 | 8.6 |  | — | — |
|  |  | B.Elbow - Wrist | 19.2 | 1.80 | 11.4 |  |  |
|  |  | Axilla - B.Elbow | 25.6 | 0.41 | 9.4 |  |  |
|  | Right ulnar nerve | Wrist - ADM | 6.32 | 3.3 |  | — | — |
|  |  | B.Elbow - Wrist | 16.9 | 1.95 | 9.9 |  |  |
|  |  | A.Elbow - B.Elbow | 19.9 | 2.1 | 23.3 |  |  |
|  |  | Axilla - Elbow | 24.2 | 0.82 | 10.5 |  |  |
| Sensory NCS | Left ulnar nerve | Dig V - Wrist | — | — |  |  |  |
|  | Left radial nerve | EPL tendon - Wrist | — | — |  |  |  |
|  | Left median nerve | Dig III - Wrist | — | — |  |  |  |
|  | Right ulnar nerve | Dig V - Wrist | 5.3 | 1.56 | 17.0 |  |  |

Description:

SCV, Sensory action potentials were not elicited in the left ulnar, radial, sural, and medial plantar nerves. Sensory conduction velocity and amplitude were reduced in the right ulnar and median nerves.

MCV, Distal motor conduction latency prolongation, reduced wave amplitude and velocity in both ulnar nerves, median nerves, and left tibial/common peroneal nerves. Wave amplitude from elbow stimulation in left ulnar and both median nerves decreased by over 50% compared to wrist stimulation.

F wave: The F-wave latency was prolonged in the left ulnar nerve, while F-waves were not elicited in the right ulnar nerve or bilateral median nerves.

Conclusion: Peripheral nerve damage in the upper and lower limbs with significant demyelination and axonal damage, and the F waves were abnormal.

**Table 2**  The standardized diagnostic criteria for CIDP in 2 children

| Criteria of EFNS/PNS 2021 | Case 1 | Case 2 |
| --- | --- | --- |
| Clinical Criteria | Relapsing, symmetric, proximal and distal muscle weakness of upper and lower limbs, and sensory involvement. their tendon reflexes in all limbs were absent.The disease course is progressive for more than 8 weeks, and relapsing-remitting. | Relapsing, symmetric, proximal and distal muscle weakness of upper and lower limbs, and sensory involvement. their tendon reflexes in all limbs were reduced.The disease course is progressive for more than 8 weeks, and relapsing-remitting. |
| Electrodiagnostic | Demyelinating polyneuropathy with secondary axonal involvement, characterized by prolonged distal latencies, markedly reduced compound muscle action potential (CMAP) amplitudes, absent F-waves, abnormal temporal dispersion, and absent sural sensory nerve action potentials (SNAPs), predominantly affecting the lower limbs | Demyelinating polyneuropathy with axonal involvement, characterized by prolonged distal latencies, reduced conduction velocities, markedly decreased CMAP amplitudes, prolonged or absent F-waves, and absent or low-amplitude SNAPs with reduced sensory conduction velocities. |
| Motor nerve conduction criteria | - Motor distal latency prolongation ≥50% above ULN in two nerves (excluding median neuropathy at the wrist from carpal tunnel syndrome), - Reduction of motor conduction velocity ≥30% below LLN in two nerves, - Prolongation of F-wave latency ≥20% above ULN in two nerves (≥50% if amplitude of distal negative peak CMAP <80% of LLN), - distal negative peak CMAP amplitude ≥20% of LLN in two nerves; or in one nerve + ≥ 1 other demyelinating parametera   except absence of F-waves in ≥1 other nerve. | - Reduction of motor conduction velocity ≥30% below LLN in two nerves, - Absence of F-waves in two nerves (if these nerves have distal negative peak CMAP amplitudes ≥20% of LLN) + ≥1 other demyelinating parametera   in ≥1 other nerve |
| Sensory nerve conduction criteria | Sensory conduction abnormalities (prolonged distal latency, or reduced SNAP amplitude, or slowed conduction velocity outside of normal limits) in two nerves. | Sensory conduction abnormalities (prolonged distal latency, or reduced SNAP amplitude, or slowed conduction velocity outside of normal limits) in two nerves. |
| Types of CIDP | Typical CIDP | Typical CIDP |
